# Making contact away from home: a bacterial secreted effector mediates inter-organelle communication

**DOI:** 10.1038/s44319-024-00312-5

**Published:** 2024-11-05

**Authors:** Rachel J Ende, Isabelle Derré

**Affiliations:** 1 Department of Molecular Biology and Microbiology, Tufts University School of Medicine, Boston, MA USA; 2 Department of Microbiology, Immunology, and Cancer Biology, University of Virginia School of Medicine, Charlottesville, VA USA

**Keywords:** Membranes & Trafficking, Microbiology, Virology & Host Pathogen Interaction, Organelles

## Abstract

A *Coxiella burnetii* effector protein acts outside the boundaries of the bacteria containing vacuole. CbEPF1 promotes membrane contact site formation between host lipid droplets and endoplasmic reticulum.

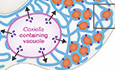

## Teaser

Inter-organelle communication via the formation of membrane contact sites (MCS) is essential for cell homeostasis. Bacterial pathogens residing in membrane bound vacuoles have exploited this biological process by secreting effector proteins that establish and function at MCS between their vacuole and host organelles. In this issue of EMBO reports, Angara et al (2024) identify a *Coxiella burnetii* effector protein, CbEPF1, that uses molecular mimicry of eukaryotic FFAT motifs to alter MCS between two host organelles: the endoplasmic reticulum and lipid droplets. The study provides the first example of a bacterial secreted effector that directly alters organelle-organelle contact away from the vacuole, identifying a novel mechanism by which bacterial vacuolar pathogens can rewire the host cell to promote infection.

Cellular homeostasis relies on inter-organelles communication. Direct organelle-organelle contact, via the formation of membrane contact sites (MCS), is essential in this process. By bringing and maintaining the membranes of two organelles in close apposition, MCS act as platforms for the efficient non-vesicular transfer of ions or lipids (Prinz et al, 2020). The endoplasmic reticulum (ER), which contacts nearly all organelles, serves as a central hub for inter-organelle communication. A family of ER-resident proteins known as VAP (VAMP-associated proteins) commonly play a role in the formation of ER-containing MCS through interaction with FFAT (two phenylalanines (FF) in an acidic tract) motif-containing proteins (Neefjes and Cabukusta, 2021). FFAT motif-containing proteins can simultaneously interact with VAP proteins on the ER and the membrane of an opposing organelle, thus bridging the two organelles.

There is increasing evidence that intracellular pathogens can manipulate cellular MCS to establish and maintain infection. Viruses target host MCS proteins and dysregulate host MCS function to facilitate viral entry, replication, and egress (Hofstadter et al, 2024). Intracellular bacteria such as *Chlamydia*, *Legionella*, and *Coxiella* reside in distinct membrane bound vacuoles that all engage in MCS with the ER (Vormittag et al, 2023). Despite different lifestyles, these bacteria have evolved common strategies to manipulate the host from within their respective vacuoles, including specialized secretion systems that deliver bacterial effector proteins into the vacuolar membrane or the host cell to target specific organelles and cellular pathways (Martinez et al, 2018). Molecular mimicry of these secreted effectors is a powerful mechanism to bring specific host factors and/or organelles in proximity of the vacuole (Mondino et al, 2020). For example, a vacuole localized *Chlamydia* effector protein displays two FFAT motifs and acts as a tether to mediate vacuole-ER MCS by interacting with VAP (Ende et al, 2022). While the role of bacterial effectors acting at MCS between the pathogen-containing vacuole and the host ER is well established, bacterial effectors that alter MCS away from the pathogen-containing vacuole remained unknown. In a new study, Angara et al, reported a *Coxiella* effector protein that contains functional FFAT motifs and rewires ER-Lipid droplet MCS.

Angara et al, screened 127 predicted *Coxiella burnetti* type 4B secretion system (T4BSS) effectors for the presence of FFAT motifs. They identified 2 FFAT motifs in the previously validated T4BSS effector CBU1370, which they renamed CbEPF1 (*Coxiella burnetti* Effector Protein with FFAT motifs 1). To determine the cellular localization of CbEPF1, the authors relied on ectopic expression in mammalian cells. CbEPF1 localized to the host ER, which may be expected based on the presence of the FFAT motifs, but also displayed a striking localization to host lipid droplets (LD). In live cell imaging, CbEPF1 initially localized to the ER at sites of LD biogenesis before gradually enriching on the surface of LD. CbEPF1 preferential localization to LD was even more pronounced under conditions that induced LD formation. Notably, CbEPF1-positive LD were extensively wrapped with the ER, as evidenced by general ER markers. Most importantly, there was a strong association of endogenous VAPA and VAPB to the surface of CbEPF1-positive LD. A similar phenotype was observed upon overexpression of MOSPD2, a VAP-family protein also known to interact with FFAT motif-containing proteins. Altogether, these elegant microscopy-based studies revealed that CbEPF1 is enriched at and facilitates MCS formation between the host ER and LD. They also implicated VAP proteins in this process.

To determine that the CbEPF1 putative FFAT motifs were functional, the authors introduced alanine substitutions in the essential position 2 of the FFAT motifs (Murphy and Levine, 2016), individually or in combination. In a series of co-immunoprecipitation experiments, CbEPF1 FFAT motifs played a redundant role in mediating interaction with endogenous VAPA and VAPB, while only one of the FFAT motifs predominantly affected binding to overexpressed MOSPD2, indicating that at least one of the FFAT motifs is required for interaction with VAP proteins. By microscopy, individual or combined FFAT motif mutations did not affect CbEPF1 association with LD, which was partially attributed to a CbEPF1 amphipathic helix. However, both FFAT motifs were necessary to induce ER wrapping around LD, indicating the functional and redundant role of the FFAT motifs in ER-LD MCS formation.

**Figure 1:**
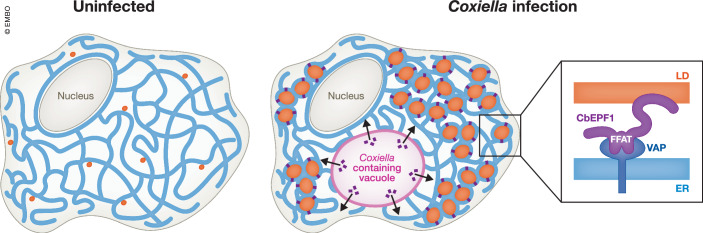
CbEPF1 mediated induction of ER-LD MCS in *Coxiella* infected cells. In uninfected cells, lipid droplets (LD, orange) form membrane contact sites (MCS) with the endoplasmic reticulum (ER, blue). In *Coxiella*-infected cells, the bacterial effector protein CbEFPF1 (purple) is secreted from the *Coxiella* containing vacuole (black arrows; pink). CpEBF1 contains 2 FFAT motifs that mediate binding to VAP proteins (dark blue) at ER-LD MCS, resulting in an increase in LD number and size.

The extensive alteration of ER-LD MCS upon CbEPF1 ectopic expression led the authors to investigate ER-LD MCS during *Coxiella* infection of macrophages. By transmission electron microscopy, the surface area of LD in close apposition to the ER increased significantly during infection. These results were in agreement with the phenotypes in CbEPF1 expressing cells and indicated that *Coxiella* promotes ER-LD MCS formation, likely through the role of CbEPF1. Given the reliance of *Coxiella* on host lipids for replication, in a final experiment, the authors investigated the effect of CbEPF1 on LD. CbEPF1 expression led to a significant increase in both the number and size of LD, with both FFAT motifs being a significant driver of LD size expansion, suggesting that via its interaction with VAP proteins and the formation of ER-LD MCS, CbEPF1 may rewire the lipid metabolism of the host cells by increasing the size of LD (Fig. 1).

The originality of the study by Angara et al, lies in the identification of the first bacterial effector protein that directly manipulates cellular MCS away from the pathogen-containing vacuole. It provides valuable insight into the diverse ways intracellular bacteria can manipulate host cells, and further supports that MCS are integral to the intracellular lifestyle of bacterial pathogens. The study also adds to the increasing number of bacterial effectors containing eukaryotic-like short linear motifs reinforcing the notion that mimicry of host proteins is evolutionary selected in pathogens as an effective strategy to manipulate host cellular pathways.

One limitation of the study is the reliance on ectopic expression of CbEPF1 due to the challenges of detecting endogenous levels of effector proteins and generating mutants in *Coxiella*. Future studies addressing these gaps will be important in determining the role of endogenous CbEPF1 during *C. burnetii* infection.

In this context, it will be interesting to investigate how CbEPF1 manipulates ER-LD MCS as well as the downstream consequences on LD and the pathogen itself. Some exciting avenues to pursue would be to determine if CbEPF1 acts as a tether, a lipid transfer protein, or both. This group previously reported the effect of *Coxiella* on LD accumulation (Mulye et al, 2018). While the authors now implicate CbEPF1 in this process, the specific role of LD accumulation on *Coxiella* growth and survival remains poorly understood. Exploring two non-mutually exclusive hypotheses—LD serving as a source of lipids and sterols and ER-LD MCS preventing the harmful accumulation of cholesterol in the ER—would shed light on the role of LD in *Coxiella* infection and host-pathogen interaction in general.

Given the striking phenotype on ER-LD MCS and LD size in response to CbEPF1 ectopic expression, the study by Angara et al could also offer a valuable tool to gain insights into LD biology.

Altogether, the study by Angara et al is a beautiful example of how through years of co-evolution with their mammalian host, intracellular pathogens have become master cell biologists from which we can learn about basic biological processes in health and disease.
